# Leg Force Control Through Biarticular Muscles for Human Walking Assistance

**DOI:** 10.3389/fnbot.2018.00039

**Published:** 2018-07-11

**Authors:** Maziar A. Sharbafi, Hamid Barazesh, Majid Iranikhah, Andre Seyfarth

**Affiliations:** ^1^Control and Intelligent Processing Center of Excellence, School of Electrical and Computer Engineering, University of Tehran, Tehran, Iran; ^2^Lauflabor Locomotion Laboratory, Institute of Sport Science, Centre for Cognitive Science, Technische Universität Darmstadt, Darmstadt, Germany; ^3^Qazvin Branch, Mechatronics Research Lab, Center of Excellence in Robotics, Islamic Azad University, Qazvin, Iran

**Keywords:** exosuit, reflex-based control, neuromuscular models, walking assistance, biarticular actuation

## Abstract

Assistive devices can be considered as one of the main applications of legged locomotion research in daily life. In order to develop an efficient and comfortable prosthesis or exoskeleton, biomechanical studies on human locomotion are very useful. In this paper, the applicability of the FMCH (force modulated compliant hip) model is investigated for control of lower limb wearable exoskeletons. This is a bioinspired method for posture control, which is based on the virtual pivot point (VPP) concept, found in human walking. By implementing the proposed method on a detailed neuromuscular model of human walking, we showed that using a biarticular actuator parallel to the hamstring muscle, activation in most of the leg muscles can be reduced. In addition, the total metabolic cost of motion is decreased up to 12%. The simple control rule of assistance is based on leg force feedback which is the only required sensory information.

## Introduction

**Figure 1:**
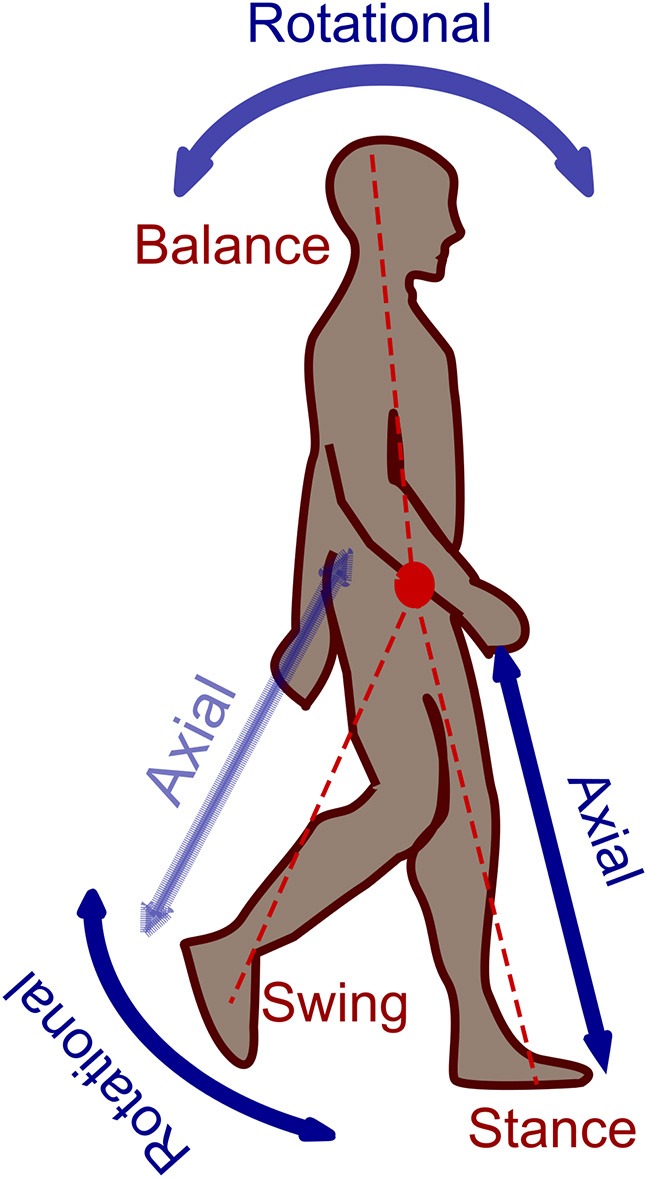
Locomotor sub-functions: Stance, Swing and Balance.

Legged locomotion is a complex nonlinear hybrid problem. There are abstract models which simplify understanding such a complex problem that can explain basic characteristics of human walking to be used for design and control of the artificial legged systems. One of the most popular concepts for abstraction is the “Template and Anchor” concept (Full and Koditschek, 1999). In this method, simple conceptual (template) models are used to describe some basic features of legged locomotion than can be extended to more detailed (anchor) models to implement on robots. Another approach is using the locomotor sub-function concept (Sharbafi and Seyfarth, 2017a) which explains legged locomotion based on three locomotor sub-functions, which are intrinsically interrelated. As shown in Figure 1, these three sub-functions are “*Stance*” for redirecting the center of mass by exerting forces on the ground; “*Swing*” as rotational movement of the free leg (no contact with the ground) around hip joint and “*Balance*” for maintaining body posture. Splitting the legged locomotion as a complex problem to three sub-problems helps us simplify understanding human locomotion (Sharbafi and Seyfarth, 2017b) and improve design, and control of legged locomotor systems (Raibert, 1986). As a result, combination of the template-anchor and locomotor sub-function concepts provide a practical tool to benefit from biological locomotor systems in design and control of robots and assistive devices (Ahmad Sharbafi et al., 2017).

There is a variety of methods to control exoskeletons inspired by human locomotion and motor control such as a force controller that behaves similar to a biological hip torque profile (Yu et al., 2014), a proportional myoelectric controller (Ferris et al., 2006), impedance control (Sharbafi and Seyfarth, 2017a), central pattern generators (CPGs) (Sobrade et al., 2017) and recently, Hybrid Zero Dynamics (HZD) method (Agrawal et al., 2017). In order to produce a proportional control signal, the myoelectric controller makes use of electromyography (EMG) of the muscles of the lower limbs for the hip exoskeleton (Ferris et al., 2006). Impedance control regarding the dynamic interactions between the leg and the ground is also very popular in exoskeletons with rigid structure like LOPES (Veneman et al., 2007).

In this paper, we focus on balance control (the third locomotor sub-function), as it might be more challenging in assisting healthy humans via exoskeletons (Full and Koditschek, 1999), in which reducing cost of transport and robustness against perturbations are addressed. Postural control consists of complex interactions between a number of systems in the human body such as musculoskeletal components, neuro-muscular synergies and adaptive mechanisms to achieve gait stability.

In Maus et al. (2010), by analyzing human and animals locomotion experiments Maus et al. showed a pattern in ground reaction forces, introducing VPP (standing for virtual pivot point) for posture control. The VPP is a point on the upper body above the center of mass at which the GRFs are intersecting during the stance phase. This observation in human walking (and animal walking and running) can be also used for posture control in models and robots (Maus et al., 2010; Sharbafi et al., 2013). A new mechanical template model was developed in Sharbafi and Seyfarth (2015) to generate VPP using an adjustable hip spring. This model which is called FMCH (force modulated compliant hip) employs the leg force to adjust hip compliance. Here, this bioinspired control approach for balancing is utilized to design and control of an exoskeleton with one biarticular actuator. This method was inspired by neuromuscular models and reflex control while it benefits from biological feedback signal. For example, Geyer et al. demonstrated that reflex-based motor control (e.g., positive feedback of muscle) can generate efficient and reliable bouncing gaits instead of using central motor commands (Geyer et al., 2003). Other studies on reflex control show the important potential of this bioinspired method for developing human gait models (Geyer and Herr, 2010; Song and Geyer, 2015) and understanding human motor control (Haeufle et al., 2012). The neuromuscular model of Geyer and Herr (Geyer and Herr, 2010) is a well-accepted human walking model, which is extended to 3d in Song and Geyer (2015) and also for analyzing performance of prosthesis and exoskeletons in Thatte and Geyer (2016). This model can be utilized as a reference neuromuscular model for assessing human (bipedal) locomotion. We use it in our simulation studies to investigate the performance of the proposed design and control approach of exoskeletons in assisting human walking.

**Figure 2:**
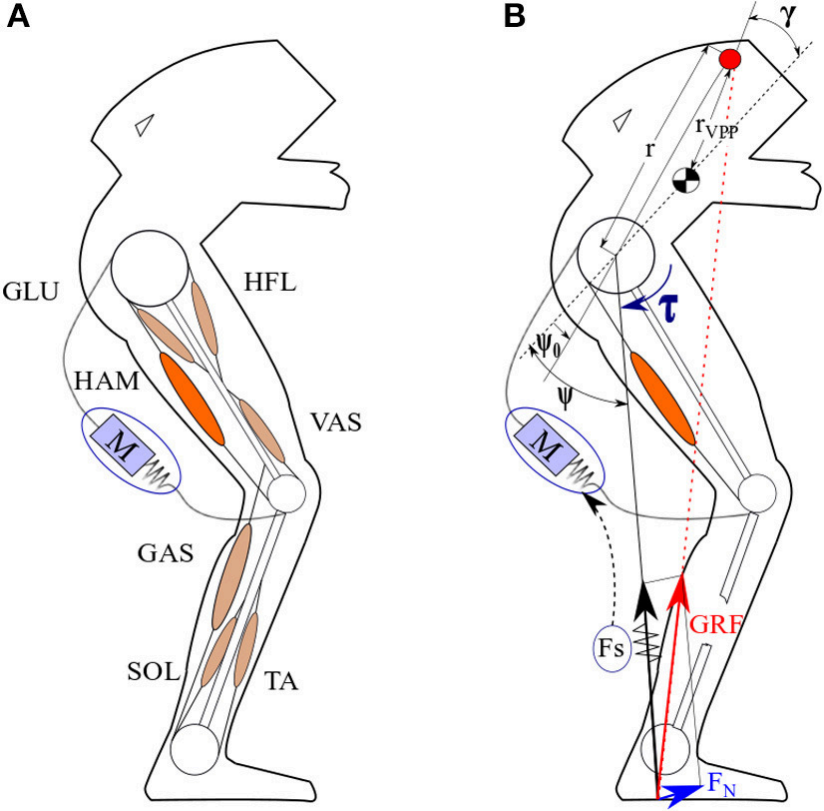
The concept of FMCH-based assistance. **(A)** The leg architecture including different muscle groups used in Geyer model (Geyer and Herr, 2010); HAM, Hamstrings; GAS, Gastrocnemius; VAS, Vastus; SOL, Soleus; GLU, Gluteus Maximus; HFL, Hip flexor; TA, Tibialis Anterior and posture control assistance with an SEA. **(B)** Implementation of FMCH-based assistance using biarticular thigh muscle.

Exoskeletons are developed to enhance human's movement capabilities, e.g., to carry heavy loads or make up for physical disorders caused by deficiencies in the muscular nervous system. Interaction between the robots and human beings can be improved if human body properties and motor control are better understood. Exoskeletons can be divided to rigid (Veneman et al., 2007; Esquenazi et al., 2012) and soft exoskeletons (Asbeck et al., 2014; Ding et al., 2017). The second group, namely exosuit, is matching better to human body properties while the rigid exoskeletons are more powerful and practical for impaired people. In exosuits, similar to the human body, the transfer of torques to the joints is performed through tensile forces parallel to the body muscles. The main application of exosuits is enhancement in performance of healthy subject needless to have powerful actuators to carry body weight. Applicability of FMCH for assisting a rigid exoskeleton (LOPES II) was shown in our previous study (Zhao et al., 2017). Here, we present advantages of implementing this method using a biarticular actuator (parallel to hamstrings, shown in Figure 2A) that can be easily implemented on soft exoskeletons.

## Methods

One of the most useful applications of studying biomechanics of legged locomotion is design and development of assistive devices. The goal of this study is to investigate the applicability (assistance level) of a bioinspired template-based method for posture control regarding reduction in metabolic cost and muscle activation. In this paper, we use the reflex-based neuromuscular model of Geyer and Herr (2010) for human-like walking. In the following, first we explain bipedal walking models and then the proposed FMCH-based control approach and its implementation as a soft exoskeleton in the aforementioned neuromuscular model.

### Conceptual modeling of bipedal walking

Bipedal walking can be described by repetition of two sequential phases: double support (DS) and single support (SS). In DS, both legs are in contact with the ground and the center of pressure (CoP) moves from hind foot to the front foot. When the hind leg leaves the ground (takeoff), SS starts and continues until its next contact with the ground (touchdown). In SS, one leg is in contact with the ground (called stance leg), while the swing leg moves to complete the step by touching the ground with a desired angle of attack.

In order to explain our control concept, a minimal model is required. To analyze and describe animal or human locomotion, simple conceptual models, called “templates,” are useful. Templates provide a great deal of information, which can help explain the features of locomotion. Templates are also used as explicit control models. One of the most useful template models for walking and running is the SLIP (Spring-Loaded Inverted Pendulum) (Blickhan, 1989; Seyfarth et al., 2002; Geyer et al., 2006). In the SLIP model, the body mass is concentrated at the center of mass (CoM) on top of a massless spring representing the stance leg.

In order to address posture control, an upper body should be added to the SLIP model. The common way is extending the model by an additional rigid trunk, resulting in TSLIP (Trunk+SLIP) model (Sharbafi et al., 2013). The basic SLIP model was developed for hopping (Blickhan, 1989) and running (Seyfarth et al., 2002). For walking a second leg is required which together with the trunk results in BTSLIP (Bipedal + TSLIP) model (Sharbafi and Seyfarth, 2015). This model is used in section Control Method Description to describe the VPP and FMCH control concepts. In the next section, a brief overview of the neuromuscular model of walking is presented.

### Human walking model

All simulations are implemented on top of a basic 2D model named muscle-reflex model (Geyer and Herr, 2010) including 7 segments (1 upper body, 2 thighs, 2 shanks and 2 feet) and 7 muscle groups for each leg. Figure 2A shows the segmentation of one leg and different muscles, implemented in this leg. The model includes muscle dynamics and hypothesized reflex pathways to generate joint torques, to mimic human walking patterns regarding kinematics, kinetics, and muscle activation. With this model, a network of muscle reflexes is utilized as a practical tool to link complex, neural circuits of biological locomotor systems and abstraction in conceptual models. By dynamic interplay of the body and the ground besides internal neural circuitry, this model can generate human-like walking which is also robust against perturbations. Furthermore, this model can be employed for assessing robots' performance in assisting human locomotion (Thatte and Geyer, 2016).

The description of the human walking model in the following is basically borrowed from Geyer et al. (2003) and Geyer and Herr (2010), where the segmented model and the reflex-based neuromuscular control are presented.

**Figure 3:**
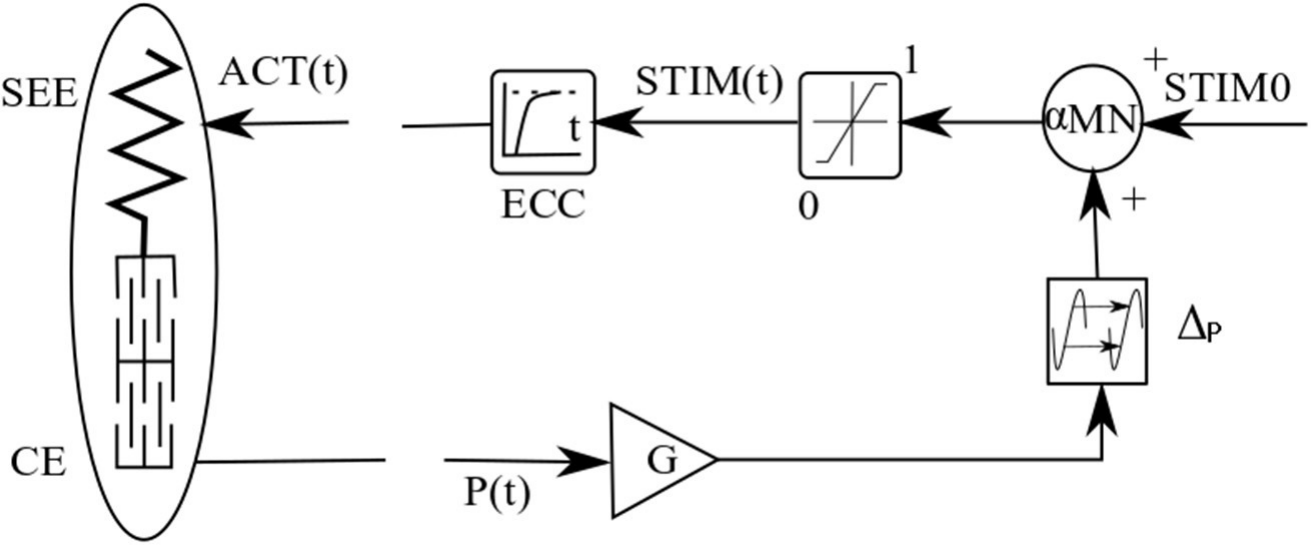
Neuromuscular reflex-based model of MTC used in walking model. The figure is adopted from Geyer et al. (2003).

In this model, MTC (muscle-tendon-complex) consists of a contractile element (CE) and a series elastic element (SEE) (Figure 3). The serial elastic element (SEE) which plays the tendon role in this model follows the nonlinear unidirectional spring, inspired by the model of van Ingen Schenau (1984):

(1) $$F_{SEE}\left(l_{SEE}\right)=\{\begin{array}{cc} {\left(\frac{l_{SEE}-l_{rest}}{l_{ref}-l_{rest}}\right)}^{2} & if\;l_{SEE}>l_{rest}\\ 0 & if\;l_{SEE}\leq l_{rest} \end{array}$$

where *l*_*rest*_ is the tendon's resting length and *l*_*ref*_ is the reference length.

The developed force of the CE is a function of the muscle activation level *A*(*t*), the maximum isometric force *F*_*max*_, the force-length function *f*_*l*_(*l*_*CE*_) (and the force-velocity relationship *f*_*v*_(*v*_*CE*_),

(2) $$\begin{array}{r} F_{CE}=A\left(t\right)F_{max}f_{l}\left(l_{CE}\right)f_{v}\left(v_{CE}\right) \end{array}$$

in which *l*_*CE*_ and *v*_*CE*_ are the muscle length and contraction speed, respectively. The force-length function is given by the following equation (Aubert, 1956):

(3) $$\begin{array}{r} f_{l}\left(l_{CE}\right)=e^{\left(c{\left|\frac{l_{CE}-l_{opt}}{wl_{opt}}\right|}^{3}\right)} \end{array}$$

Where _*l*_*opt*__, *w* and *c* are the optimum CE length, the width of the bell-shaped *f*_*l*_(*l*_*CE*_) curve and a constant value, respectively. The force-velocity relation is composed of the Hill model (Hill, 1938) and Auber model (Aubert, 1956) for contraction and protraction, respectively (see Geyer et al., 2003, for details):

(4) $$f_{v}\left(v_{CE}\right)=\{\begin{array}{l} \frac{v_{max}-v_{CE}}{v_{max}+Kv_{CE}}\;\;\;,\;v_{CE}<0\\ N+\left(N-1\right)\frac{v_{max}+v_{CE}}{7.56Kv_{CE}-v_{max}},\;v_{CE}\geq 0 \end{array}$$

in which *v*_*max*_ and *N* denote maximum contraction velocity and a constant value, respectively. The activation is resulted from stimulation signal, which uses reflex pathway as shown in Figure 3.

(5) $$\begin{array}{r} \tau A\left(t\right)=STIM\left(t\right)-\frac{d\left(A\left(t\right)\right)}{dt} \end{array}$$

(6) $$STIM(t)=\{\begin{array}{c} \;STIM0,\;t<\Delta_{p}\\ STIM_{0}\pm GP\left(t-\Delta_{p}\right),\;t\geq \Delta_{p} \end{array}$$

where *STIM*(*t*), *A*(*t*), *STIM*_0_ and are stimulation signal, activation signal, stimulation bias and signal propagation delay, respectively.

The feedback sensory signals *P* is given by a combination of three reflex pathways CE length (*l*_*CE*_), velocity (*v*_*CE*_), and the muscle force (*F*_*CE*_) which is equal to the MTC force and the *F*_*SEE*_. In this model, different combinations of reflex pathways are considered for different muscles and the gain values are optimized to achieve stable human-like walking.

In order to show the assistance level of the exosuit, we compare the metabolic cost (see section Optimization) for three different models. First, we consider the human walking model without assistance, borrowed from Geyer and Herr (2010). In our simulations, all the parameters of the neuromuscular model are set to values defined in Geyer and Herr (2010). Table 1 shows the muscle properties and reflex gain factors and the model can be downloaded here.

**Table 1:** Muscle properties and reflex gains in the neuromuscular model of human walking, from Geyer and Herr (2010).

**Muscle**	**Gain factor**	***F*_*max*_ (*N*)**	**$v_{max}\left(l_{opt}s^{-1}\right)$**	***l*_*opt*_ (*cm*)**
HFL	0.35	2,000	12	11
GLU	0.4	1,500	12	11
HAM	0.65	3,000	12	10
GAS	1.1	1,500	12	8
VAS	1.15	6,000	12	5
SOL	1.2	4,000	6	6
TA	1.1	800	12	4

In the second model, we added exosuit for assistance, neglecting the additional weight of the exo. The parameters of the exo are found using optimization techniques explained in section Optimization. This model can represent the level of assistance compared to the transparent mode. Another application of this model is to represent walking of a human subject wearing a passive version of the assistive device (under construction) with low weigth to compare with normal walking. In addition, in most of the studies on exosuits, the weight of the actuation setup is neglected using the tethered actuation system in which the actuators and the corresponding electronics are mounted separately on a fixed frame and forces are transferred through cables connected to human body by ligth wearable parts (Asbeck et al., 2014; Ding et al., 2017). However, in the third model, we consider additional weight of the active exosuit including the motors, sensors, electronics and the box placed in the backpack besides the passive elements such as the cables, springs, wearable parts. In this model, we have added 4 kg to the mass of the upper body based on the parameters of our recently developed exosuit shown in section Discussion.

### Control method description

As the focus of this study is on assistance of healthy subjects to enhance their motion performance by exosuits, balance control is more crucial. For impaired people or elderly people who use crutches, stance and swing sub-functions have more importance. In this section, we explain the VPP and FMCH concepts for balance control and how to benefit from biarticular actuation to employ the FMCH on muscle-reflex model for walking assistance. Thus, first we explain VPP and FMCH concepts based on BTSLIP model that include prismatic (one-segment) leg, and then show how FMCH method can be implemented on muscle-reflex model that include segmented leg.

#### Balance control, VPP and FMCH

Humans walk with upright upper body as one of the main differences with other animals (Maus et al., 2010). Because of inherently unstable dynamics of the inverted pendulum, which is a popular model of balancing in bipeds, keeping upright posture is more challenging than body posture in multi-legged models. However, using external support, postural stability becomes less critical. Therefore, vertical body alignment, which has a key role in stabilizing human locomotion can be handled in an easy way. This external support can be considered as the core idea of virtual pivot point (VPP) concept (Maus et al., 2010).

As mentioned in section Introduction, it is observed that the ground reaction force (GRF) vectors of the stance leg in humans (and animals) walking intersect at a point on the upper body above CoM (Maus et al., 2010). This intersection point, which is called VPP can translate the balancing from an inverted pendulum model to a virtual pendulum (VP) as a point mass at CoM hanging from the VPP. This concept can be used for posture control of bipedal gaits (see Figure 2B). Using the BTSLIP model, a hip torque (τ) between upper body and the virtual leg (connecting the hip to the CoP) can be found to redirect the GRF going through a determined VPP (Sharbafi and Seyfarth, 2015).

(7) $$\begin{array}{r} \tau =F_{s}l\frac{r_{h}sin\psi +r_{VPP}sin\left(\psi -\gamma \right)}{l+r_{h}cos\psi +r_{VPP}cos\left(\psi -\gamma \right)} \end{array}$$

where τ, *F*_*s*_, *l*, ψ, *r*_*VPP*_and *r*_*h*_ are the hip torque, leg force, leg length, hip angle, the distance from CoM to VPP and from VPP to hip joint, respectively. The VPP angle is defined by γ as the angle between body axis and the vector from CoM to VPP as shown in Figure 2B. It is important that for posture control using VPP concept we do not need to measure the trunk angle with respect to ground and just the internal angle between upper body and the virtual leg is sufficient. Therefore, a hip spring between upper body and the leg in BTSLIP model can be used to measure this angle. In the next section, we depict how biarticular thigh muscles can be used to measure this angle. The second significant point is about modulation of the mentioned hip spring by the leg force (*F*_*s*_) in Equation (7). Based on these two concepts the FMCH model was developed (Geyer et al., 2003) which simplifies the posture control as follows.

(8) $$\begin{array}{r} \tau =k_{h}F_{s}\left(\psi_{0}-\psi \right) \end{array}$$

In which *k*_*h*_ and ψ_0_ are the normalized stiffness and the rest angle of the adjustable hip spring, respectively. It was shown that for a range of joint angles variations, used for human normal walking, FMCH presents a very precise approximation of VPP (Sharbafi and Seyfarth, 2015). As it is shown in Figure 2, the GRF vector can be decomposed to the perpendicular and axial directions. To control upper body posture using VPP method we can adjust Perpendicular GRF by hip torque, such that GRF direction crosses VPP. However, when we use neuromuscular model of Geyer and Herr (Geyer and Herr, 2010) with segmented legs, the model needs to be extended. For this we define the virtual leg from hip to ankle and the virtual hip torque between the upper body and the virtual leg. To control this virtual hip torque both hip and knee joints should be controlled in coordination. In the following, we demonstrate how biarticular muscles with appropriate lever arm ratios can be employed to provide access to control the virtual hip torque and then, the perpendicular term of the GRF with respect to the virtual leg.

#### Segmentation and biarticular actuation

One of the important characteristics of the human body, which is beneficial for efficient locomotion, is the leg morphology. The zigzag configuration of the human leg is opposite to birds' leg curvature. Each of these configurations is optimized due to the body properties (e.g., position of CoM with respect to the hip, the ratio between different leg segments' lengths) during million years of evolution. For example, the segment lengths ratio in human leg correspond to the required highly loaded MTC in the human leg, determined by the stress-strain properties of the tendons (Seyfarth et al., 2000).

One of the most important properties of muscular systems in animals (including humans) is using biarticular muscles. The neuromuscular model of Geyer includes two biarticular muscles (HAM and GAS) and five monoarticular muscles GLU, HFL, VAS, TA and SOL as shown in Figure 2A. Several advantages of using biarticular muscles beside monoarticular ones were depicted such as coupling of joint movements, velocity contraction, passive energy transfer between and homogenous bending of the adjacent joints (van Ingen Schenau et al., 1990; Seyfarth et al., 2001). In our previous studies, we have shown the significant contributions of biarticular muscles to different locomotion sub-functions control (Sharbafi et al., 2016a). It was shown that with appropriate design of the thigh biarticular actuators in BioBiped3 robot, GRF direction can be controlled with minimum interference to GRF magnitude. In addition, minimizing the influence of GRF direction control on the axial leg function results in a decoupled control of stance and balance locomotor sub-functions. As posture control using the VPP concept is based on GRF direction control, we design our soft exo by a compliant adjustable biarticular actuator parallel to the human HAM muscle.

The ratio between lever arms of the two connected joints is a key design parameter in biarticular actuation. Due to similar size of shank and thigh in human leg, setting hip to knee lever arm ratio to 2:1 minimizes the crosstalk between changes in axial and perpendicular GRF (Sharbafi et al., 2016a). Therefore, here we attach a compliant actuator connecting the upper body to the shank, while the moment arm at hip is twice the moment arm at knee joint (see Figure 2B). Then, the FMCH control approach is employed to adjust stiffness in which the actuator is modeled by an adjustable spring with length*l*^*exo*^, rest length $l_{0}^{exo}$ and normalized stiffness*c*_*h*_. Therefore, the actuator force *F*^*exo*^is calculated as follows

(9) $$\begin{array}{r} F^{exo}=c_{h}F_{s}max\left(l_{0}^{exo}-l^{exo},0\right)=c_{h}F_{s}max\left(\Delta l^{exo},0\right). \end{array}$$

The *max* function in this equation shows that the actuator is unidirectional. Therefore, the actuator can pull the segments (like biological muscles or the SEA, shown in Figure 2). Considering the hip to knee muscle lever arm ratios of 2:1, the actuator length change will be proportional to the variation in the angle between the upper body and the virtual leg (ψ in Figure 2B).

(10) $$\begin{array}{r} \Delta l^{exo}=r_{hip}^{exo}\Delta \psi . \end{array}$$

in which $r_{hip}^{exo}$ is the actuator lever arm at hip joint and . Derivation of this equation is presented in the Appendix.

As a result, the muscle force is given by

(11) $$\begin{array}{r} F^{exo}=c_{h}F_{s}r_{hip}^{exo}max\left(\Delta \psi ,0\right)=k_{h}F_{s}max\left(\psi_{0}-\psi ,0\right) \end{array}$$

This equation is similar to the FMCH control for BTSLIP model, explained in Equation (8). This shows that using biarticular actuator with hip to knee lever arm ratio 2:1, precise implementation of VPP is achieved through FMCH model. Based on this argumentation, we suggest designing an assistive device (e.g., in soft-exo) to generate forces almost parallel to the HAM muscle. This exo generates the following hip ($\tau_{hip}^{exo})$and knee ($\tau_{knee}^{exo})$ torques

(12) $$\begin{array}{r} \tau_{hip}^{exo}=2\tau_{knee}^{exo}=r_{hip}^{exo}F^{exo}. \end{array}$$

This method was implemented on LOPES II robot by emulating biarticular actuator using two monoarticular hip and knee actuators (Zhao et al., 2017).

#### Optimization

In the proposed control approach (11), *k*_*h*_ and ψ_0_ are the two tuning parameters of the controller. First of all we need to define stable walking. Here we use step-to-fall approach to detect stable gaits. The model is initiated with a specific initial condition adopted from Geyer and Herr (2010) for normal walking without assistance. Then, the stability is verified if the model can take 50 steps. In order to minimize energy consumption in human body (metabolic cost), we define the following cost function (*J*) for optimization.

(13) $$\begin{array}{r} J=\frac{1}{M}\sum_{i=N-M+1}^{N}\frac{E_{met}^{total}}{d}. \end{array}$$

This metric shows the average of consumed energy for traveling 1 meter in the last *M* steps. In our simulations, *M*and *N* are set to 30 and 50 determining the mean value of the metabolic cost for the last 30 steps of 50 steps defined for a stable solution. Here, we considered 20 steps to pass the transient behavior and to reach the steady state. In this equation, *d*is the travelled distance and $E_{met}^{total}$ denotes total metabolic cost of human walking ($E_{met}^{total}=\overset{7}{\underset{i=1}{\sum }}E_{met}^{i}$), consumed by the seven different muscle groups defined in the neuromuscular model (Geyer and Herr, 2010; see Figure 2). For each muscle the metabolic cost is calculated as follows

(14) $$\begin{array}{r} E_{met}=\int_{t1}^{t2}P_{met} \end{array}$$

where *P*_*met*_(*t*) is instantaneous metabolic power (Krishnaswamy et al., 2011). This value is computed for each muscle and their summation gives the total metabolic cost of the whole body motion ($E_{met}^{total}$). At any time *t*, *P*_*met*_(*t*) is obtained as follows

(15) $$\begin{array}{r} P_{met}\left(t\right)=p\left(v_{CE}/v_{max}\right)\times A\left(t\right)\times |F_{max}\times v_{max}| \end{array}$$

in which *p*(*x*) is a function approximated based on empirical data (Alexander, 1997) by

(16) $$p\left(x\right)=\{\begin{array}{c} 0.01-0.11\left(x\right)+0.06exp\;\left(23x\right),\;x<0\\ 0.23-0.16\exp\left(-8x\right)\;,x\geq 0 \end{array}$$

For this, we implemented an optimization procedure in the neuromuscular model (presented in section Human Walking Model). To find the optimal values of *k*_*h*_ and ψ_0_ that minimize the normalized metabolic cost [defined by (13], we searched in definable ranges of these parameters. These ranges are 0 to 10 for the normalized stiffness *k*_*h*_ and −25 to 25° for the rest angleψ_0_. Out of these ranges is not obtainable due to limitations in actuation mechanism. Here, we assume that the body control parameters including the reflex gains are fixed as presented in Geyer and Herr (2010). Hence, addition of the biarticular soft-exo can only affect the muscle force generation though changing the reflex signal (e.g., muscle forces). It is clear that optimizing the parameters of both soft-exo and reflex gains will result in higher reduction in metabolic cost as the fixed gains are one parameter set in the gain parameter space that may have other minima with lower metabolic costs. Hence, our method can result in higher assistance if we consider human adaptation to the assistive device.

## Results

In this section, we explain the simulation results of applying the FMCH controller on a biarticular thigh actuator to assist human walking at normal walking speed (1.3 m/s). We compare muscle forces, activations and metabolic costs in the different 3 models. The first one is for human walking without assistance; the second one is with assistance in an ideal case without addition of the exoskeleton mass. This demonstrates the quality of the proposed method regardless the implementation issues. Finally, a *4 kg* package is considered on the upper body to contain two actuators, electronics and processor. The force is transferred through a cable drive mechanism similar to the soft-exos (Ding et al., 2017). The kinematic behavior of the gait and the motion speed are not significantly different (less than 5%) in the three models (not shown).

The optimal control parameters for the stiffness and the rest angle of the exo based on Equation (11) are *k*_*h*_ = 5.65 and $\;\psi_{0}=-7^{{}^{\circ}}$. These numbers show that during the stance phase, the exosuit starts to pull after touchdown until ψ reaches −7° which is slightly after mid-stance. In the swing phase, the exo does not produce any force because the leg force is zero.

**Figure 4:**
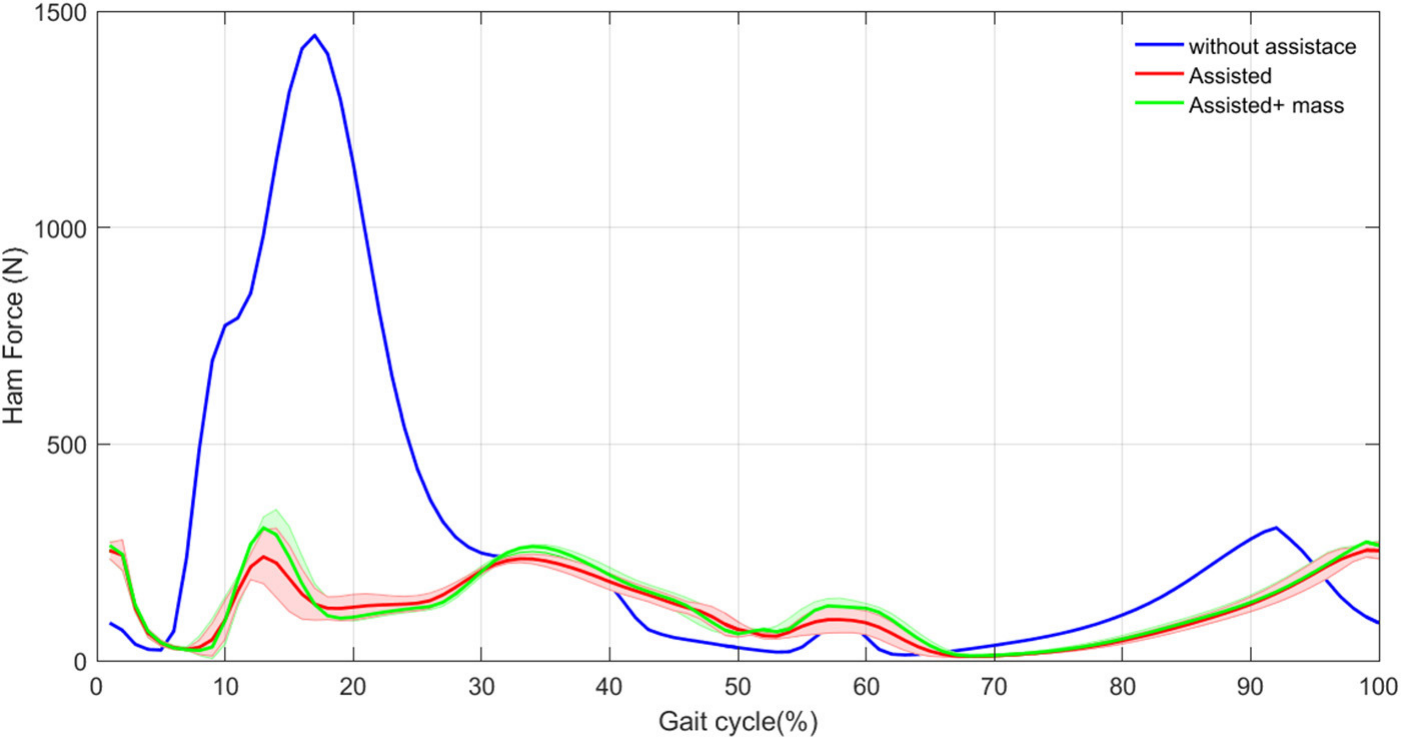
HAM muscle mean force (thick curves) and standard deviation (shaded areas) with and without assistance in 30 steps.

**Figure 5:**
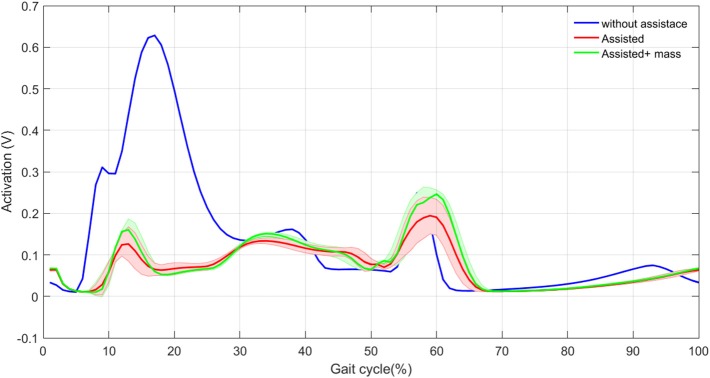
HAM muscle activation mean (thick curves) and standard deviation (shaded areas) with and without assistance in 30 steps.

In the following the muscle force, activation and metabolic power are compared to demonstrate the advantages of the proposed method for design and control of the exoskeleton. Similar to previously explained approach for the cost function (section Optimization), here we consider the last 30 steps and the mean and standard deviation are shown in the following figures. First, in Figures 4, 5 we show the values for the Ham muscle, as it is parallel to the actuator. Then the mean values for one stride are shown for different muscles. In sectiion Energy Economy, the total metabolic power is used to realize the effectiveness of the proposed technique. Finally, the contribution of the exosuit design on posture control is analyzed using VPP.

### Effects on HAM muscle

In this section, we show the activation and the force produced by HAM muscle in the three different cases. Figure 4. Illustrates the mean and standard deviation of the HAM muscle force in 30 steps, without assistance compared with these values with assistance. As expected, the force modulated compliant hip controller produces a significant part of the required force of HAM during stance phase. As a result, considerable decrease in HAM force is observed in the first 30% of the gait cycle. In spite of zero contribution of the exo in the swing phase, forces differ due to the effects from the stance phase and also the second leg, which is in stance phase. In the original model (without assistance) the variance among 30 steps is close to zero. Although, addition of the exosuit changes the force patterns slightly from step to step, the variance is still negligible.

Figure 5 shows the activation signals for the HAM muscle. The activation patterns are similar except in the first 30% of the gait. In this period, the exosuit generates most of the required efforts resulting in activation reduction in HAM muscle. These results are in line with the observations in Figure 4 for developed force. Addition of the exo mass does not have significant effects on the HAM muscle activation and force. This means that most of the required force in HAM to compensate the exo mass is provided by the actuator. Therefore, the person who wears the exosuit will not suffer from the additional mass of the robot. In addition, variance in the activation signal increases by adding the exo, which is similar to the previous observations in Figure 4. The only difference appears in the beginning of the second step when the variance is larger in contrast to the force patterns. This might relate to pushoff in which HAM muscle activation is influenced by variations in other muscles at each step, but this is not significantly reflected in muscle force. Generally speaking, reduction in HAM muscle force and activation in the first 30% of the stride can be considered as the main effect of exosuit contribution to the stance leg assistance and even without adaptation of the reflex gains, a periodic motion (with low variance) can be obtained after addition of the assistive device. This second outcome might relate to the bioinspired control principle employed in design and control of our exosuit.

### Effects on the whole leg neuromuscular control

**Figure 6:**
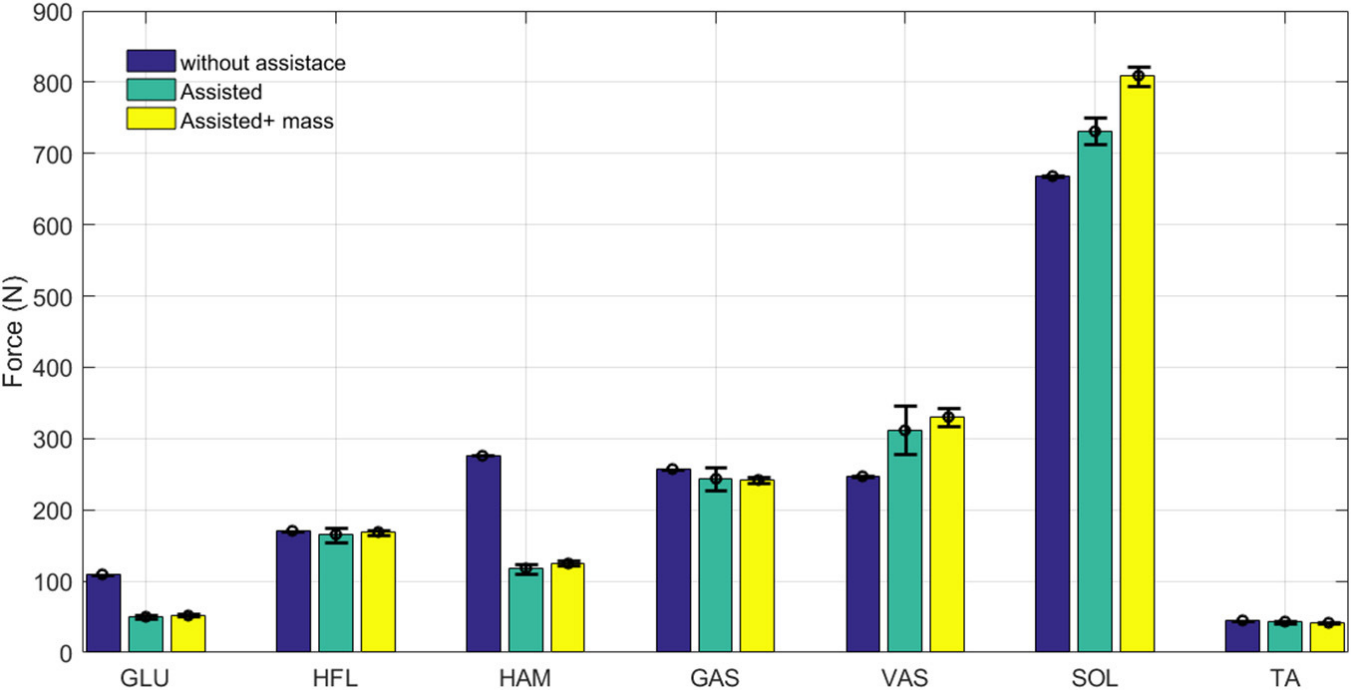
Grand mean muscles forces during one stride with and without assistance. Error bars represent ±1 standard deviation in 30 steps.

**Figure 7:**
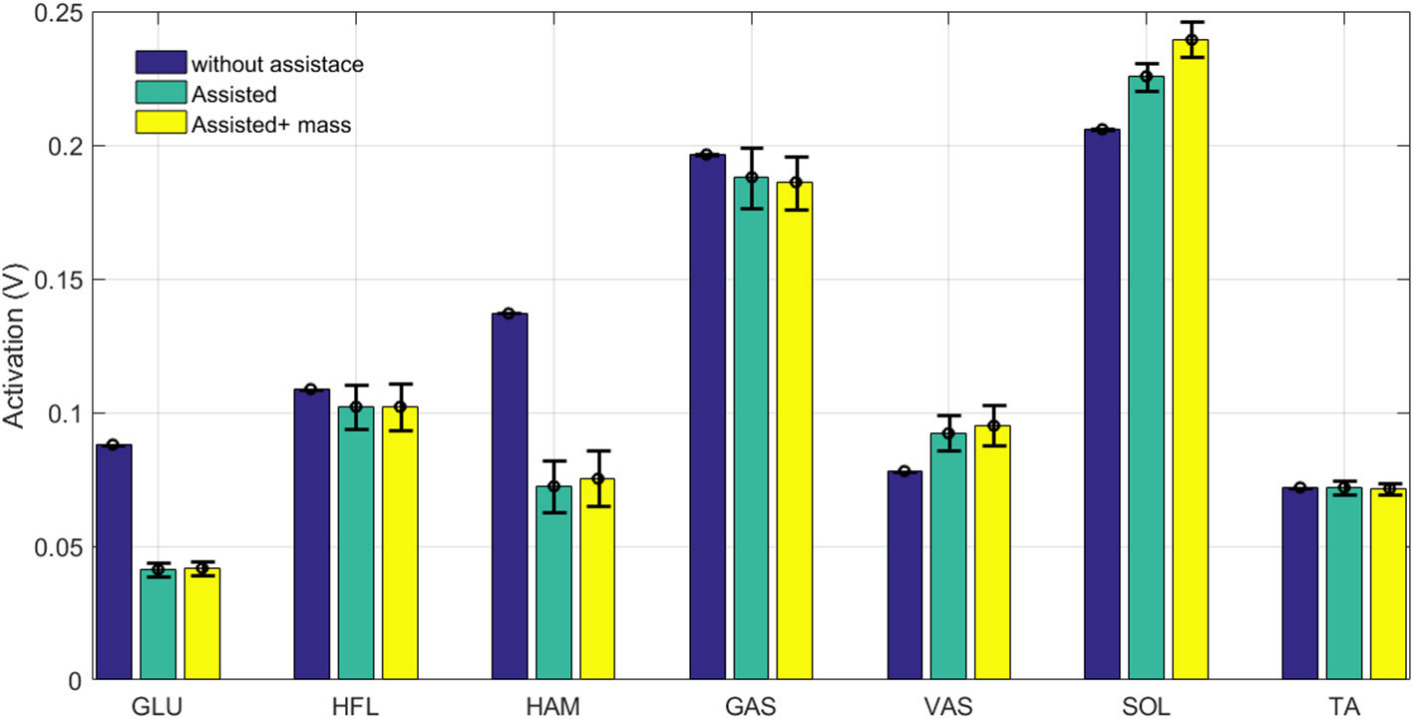
Grand mean muscles activation during one stride with and without assistance. Error bars represent ±1 standard deviation in 30 steps.

Based on the reflex control in the neuromuscular model, assisting hip biarticular muscles influences activation and force generation in the other muscles. In this section, we analyze these effects using the grand mean as the average of the mean values of the last 30 steps (gait cycles). Accordingly, Figures 6, 7 depict the grand mean of force (*F*_*GM*_) and activation signals (*A*_*GM*_) for different muscles during 30 steps. In addition, the standard deviations among different steps are shown in the same figures. The grand mean and standard deviation of the muscle forces are calculated as follows.

(17) $$\begin{array}{rcl} F_{GM} & = & \frac{1}{30}\sum_{i=21}^{50}{\overset{︷}{\frac{1}{T_{i}}\int_{0}^{T_{i}}F_{i}\left(t\right)dt}}^{\bar{F_{i}}} \end{array}$$

(18) $$\begin{array}{rcl} F_{Std} & = & \sqrt{\frac{1}{30}\sum_{i=21}^{50}{\left(\bar{F_{i}}-F_{GM}\right)}^{2}} \end{array}$$

in which subindex *i* denotes the *i*^*th*^ step. Similar equations are utilized to calculate the grand mean and standard deviation for activation signal of each muscle.

As expected, both force and activation are reduced in hip extensor muscles (HAM and GLU). Activation reduction in the other three muscles (GAS, HFL, and TA) does not significantly change the muscle force. This results in lower metabolic cost in these muscles while generating similar forces. Therefore, assisting the HAM muscle can reduce energy consumption in these muscles without significantly changing their developed forces. Although these muscles are more responsible for balancing, they also contribute to axial leg function. As a result, increases in monoarticular knee and ankle extensors (VAS and SOL) are observed. The additional mass of the exo-suit should be also handled by growth in SOL and VAS forces. As shown in these figures, the results are quite consistent for all muscles, as the standard deviations during 30 steps are very small. Adapting reflex gains after adding the exo (not performed in this study) may result in even smaller variance similar to the first case (blue bar) without assistance.

### Energy economy

**Figure 8:**
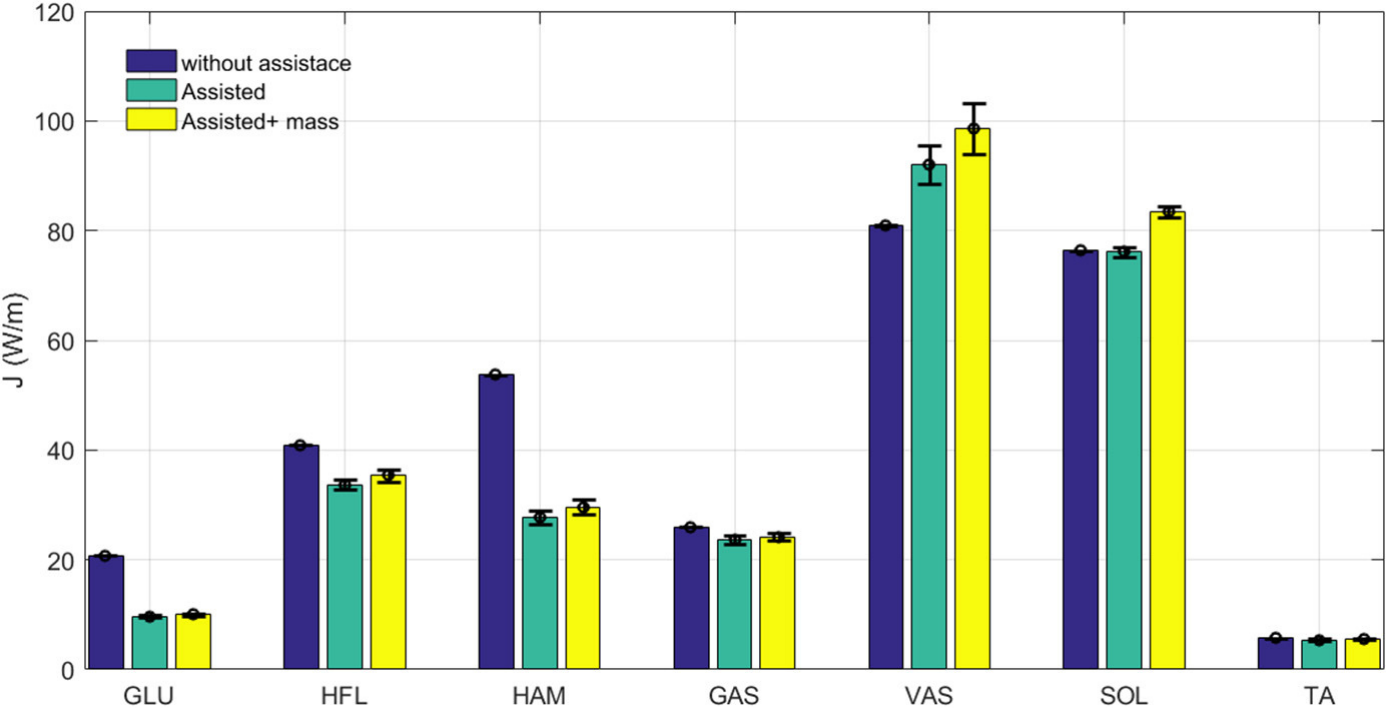
Grand mean muscles Metabolic cost during one stride with and without assistance. Error bars represent ±1 standard deviation in 30 steps.

To investigate the effect of variations in activation and force of different muscles on the energy consumption, the normalized metabolic cost [calculated by (13)] of individual muscles are compared in Figure 8. In comparison between unassisted walking and the ideal assisted model (without additional mass), metabolic effort is just increased in one (VAS) muscle. In addition, the reduction in energy consumption of HAM, HFL, and GLU dominate the increment in VAS. Similar to the force and activation behavior, additional mass is mainly reflected in the growth of energy consumption in SOL and VAS. Still, decreased metabolic cost in other muscles is significantly higher than extra energy, required to support the additional mass.

**Figure 9:**
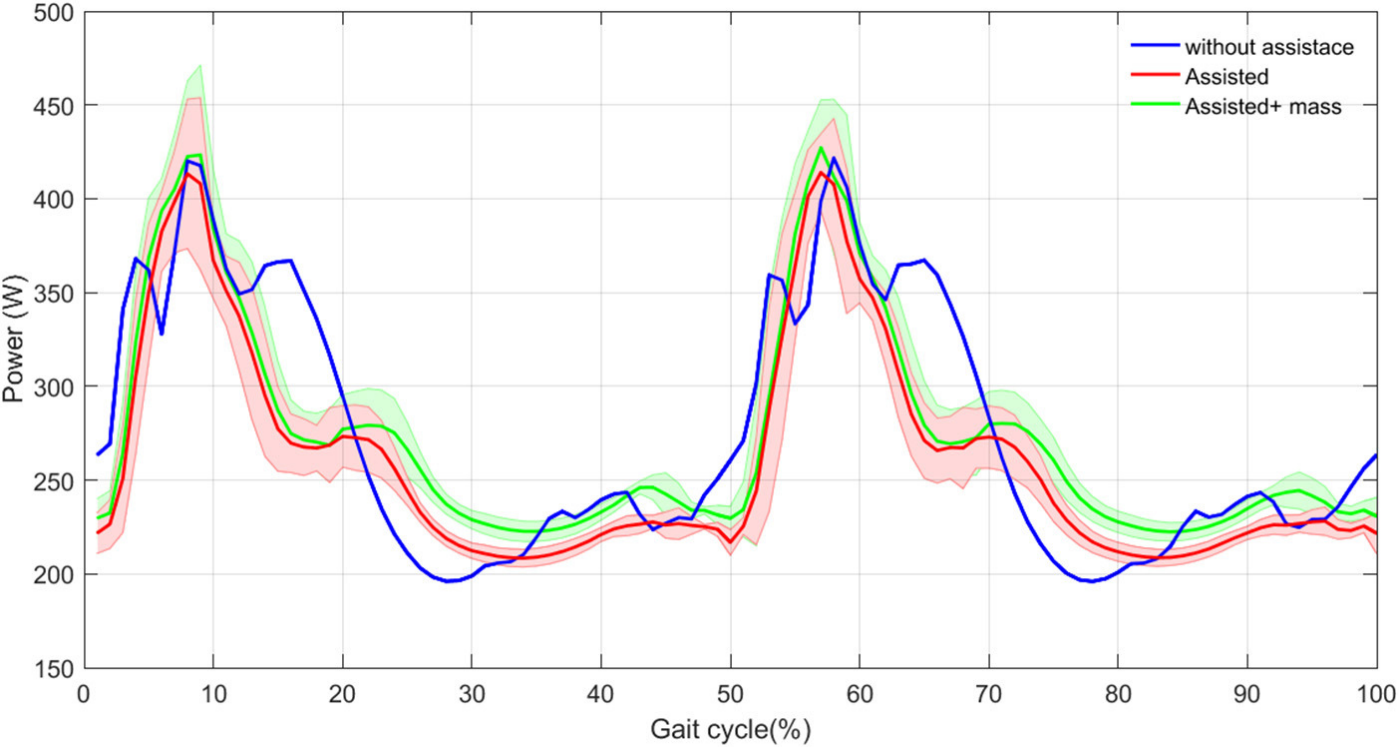
Total metabolic power mean (thick curves) and standard deviation (shaded areas) with and without assistance in 30 steps.

To investigate the level of assistance at different moments of the gait cycle, the total metabolic power during a complete stride is drawn in Figure 9. This graph shows the mean and the total metabolic power of both legs, in a complete stride among 30 last steps. Considerable reduction in metabolic power is observed in the first 20% of the gait cycle meaning that the exosuit supports walking until shortly before the midstance. This is coincident with the time slot that the HAM muscle contributes the most. As the exo actuator is parallel to the HAM muscle, its contribution is low (or even zero) after midstance (more precisely when ψ < ψ_0_ = −7°). It is also observed that the three-hump pattern of the metabolic power in the unassisted case is changed to a single-hump resulting in significant reduction in metabolic power. Although an increase in metabolic power consumption is observed around midstance, it is compensated afterward (about 33% of the gait cycle). Roughly speaking, the total energy from midstance to touch down of the next leg is almost constant with and without assistance. Hence, after the exo contribution in first 20% of the gait cycle the metabolic power does not change significantly in the ideal model of assisted case, compared to the unassisted case while addition of the exo mass increases the metabolic power in this period. Interestingly, the human power consumption is barely affected by additional mass in the first 20% of the gait cycle (before midstance). It means that the exo compensate the required energy to support the additional mass. However, this cannot continue until the end of the gait cycle, because of the reduction in assistive device contribution. Therefore, the total reduction in metabolic cost will be lowered. Similar pattern is observed in the second step and the total metabolic energy is reduced with assistance.

Comparing the total energy consumption per stride, demonstrate advantages of walking assistance. As a result, the reductions in the model with and without exo mass are 6 and 12%, respectively. These numbers are 48 and 45% for the HAM muscle. After assistance, the motion performance is kept, except negligible reduction in the walking speed. Reduction in total metabolic cost using an exosuit with just one actuator for each leg that supports the body only in stance phase of the corresponding leg is considerable.

**Figure 10:**
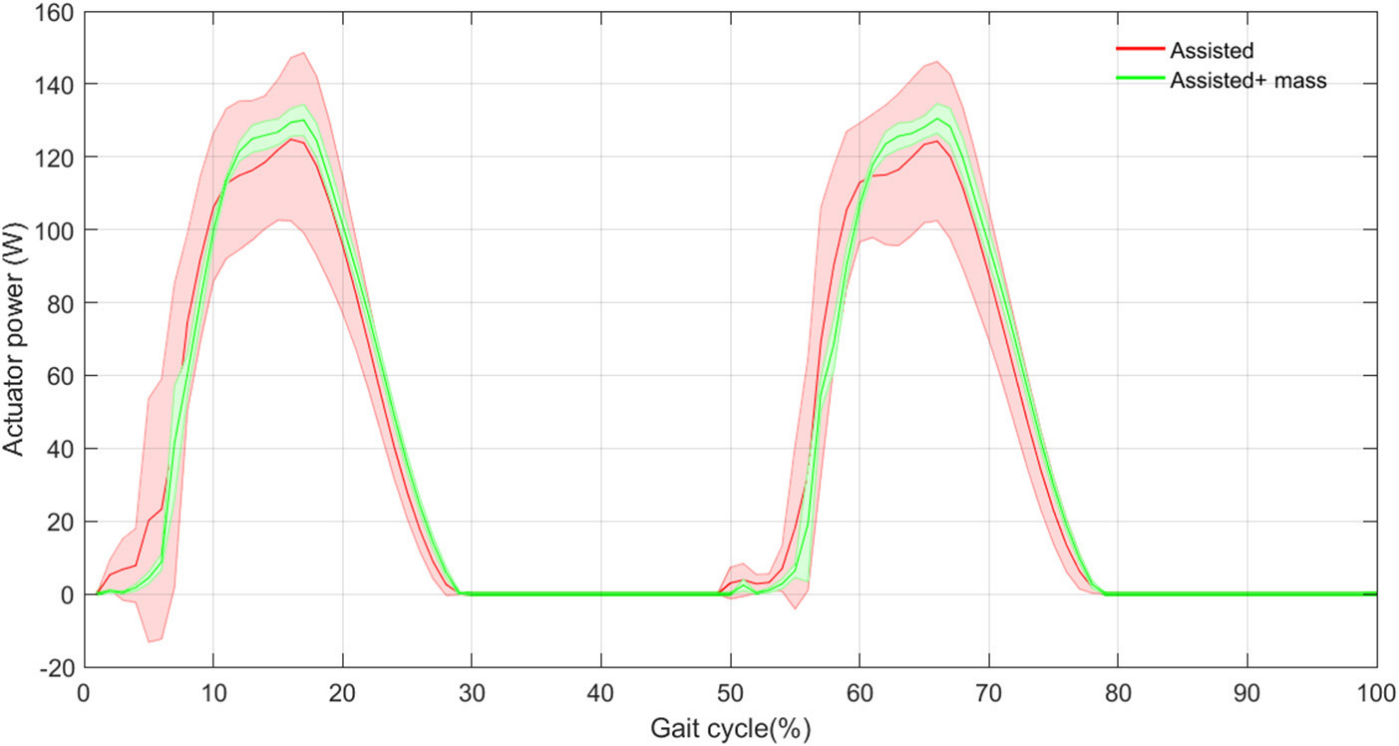
The actuator power mean (thick curves) and standard deviation (shaded areas) with and without assistance in 30 steps.

In order to validate the effectiveness of the proposed method, in Figure 10, we show the power consumption in the exosuit during one stride. Here, we have assumed that the FMCH is implemented by a rigid actuator, which mimics the adjustable compliance. Therefore, this is the maximum power required for implementing the control concept. Obviously, benefitting from compliant structure (e.g., by designing a variable impedance actuator with parallel compliance) can significantly reduce the required power of the actuator. Nevertheless, it can be shown that there is a big advantage in the proposed design and control. The results shown in Figure 10 support the previous observations in Figure 9. Note that in the proposed exo design and control, the assistive device contributes in half of the gait cycle in which the leg is in stance phase and the so called biarticular compliant element is stretched. Interestingly, the variance in power generation among 30 steps is reduced by adding the exo mass.

### Balance control

**Figure 11:**
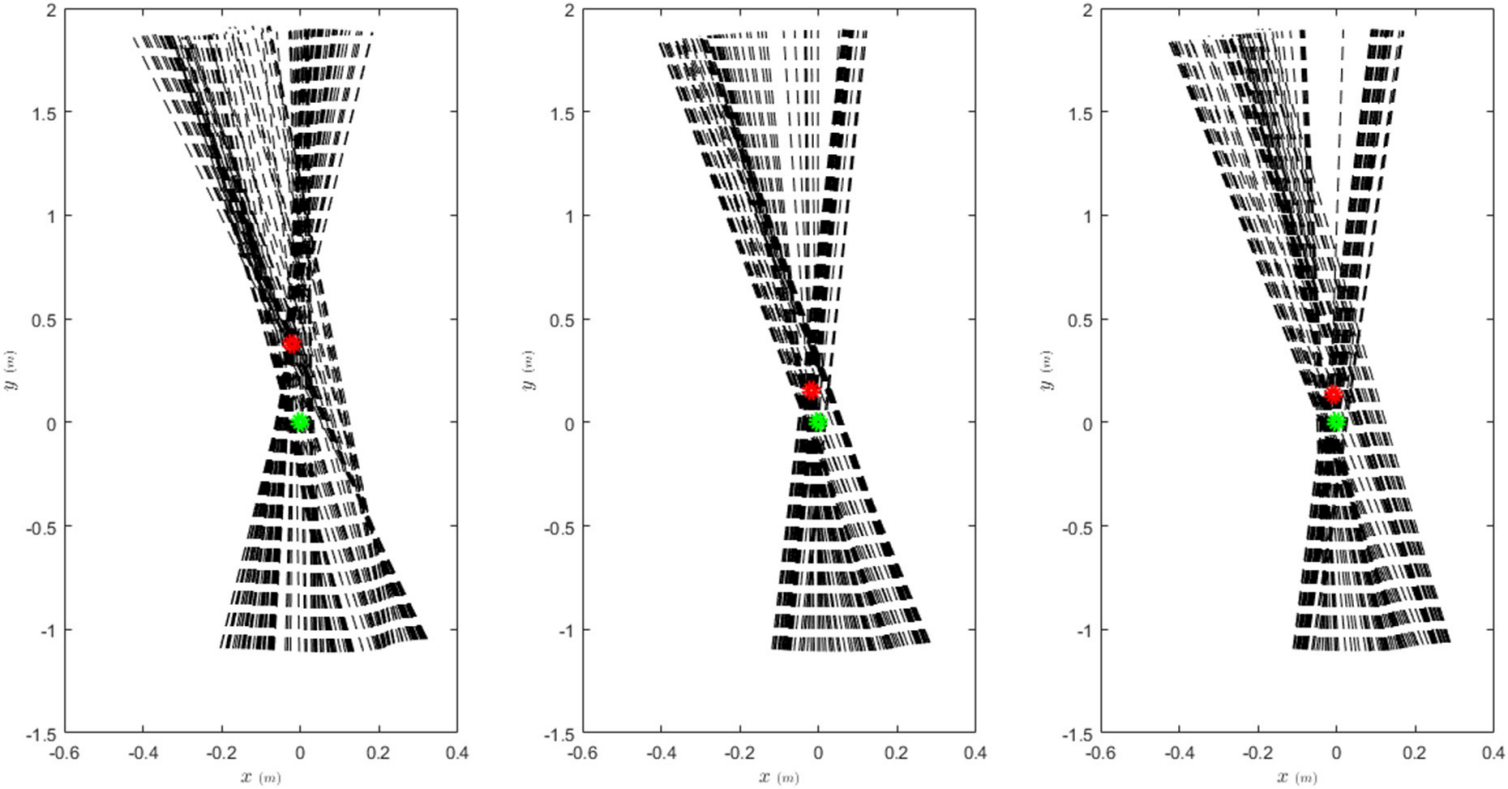
Ground reaction forces in body coordinate frame (dashed black lines) and the VPP (red circle) with and without assistance. The body coordinate frame is centered at CoM (green circle) with vertical axis aligned with the upper body orientation.

As the core control concept of FMCH is the VPP model that is introduced regarding posture control, here we demonstrate the effects of walking assistance on balancing. In Figure 11, the GRF (ground reaction forces) are demonstrated in the coordinate frame centered at CoM (center of mass) with vertical coordinate aligned with upper body orientation. For regular walking without assistance, the VPP exists about 40 cm above CoM which is in line with finding in human walking at moderate speeds (Maus et al., 2010). By assistance of the exo, the GRF vectors are more focused which support balance control through VPP concept. In addition, the VPP becomes closer to CoM [smaller *r*_*VPP*_ in Equation (7)] while the VPP angle (γ) increases. This means more less oscillations in upper body and more performant posture control. This behavior is slightly deformed by adding the exo mass. In general, addition of the exosuit clearly improves posture control as expected.

## Discussion

The bioinspired design and control of a soft exoskeleton was proposed in this paper. Concentrating on balancing as one locomotor sub-function, two basic design principles were employed: (i) As a control design principle, leg force is used as a sensory feedback signal to adjust hip compliance (FMCH) (Sharbafi and Seyfarth, 2015). This approach is motivated by the VPP concept for posture control (Maus et al., 2010). (ii) A second design principle is employing biarticular actuators to simplify GRF direction control and consequently, posture control. Using this actuator-skeletal design principle, we can benefit from synchronization between hip and knee joint and increasing efficiency by transferring energy between joints. Using these bioinspired principles we can improve interaction between human and the assistive device. Instead of regular position, force or impedance control of the end effector, in our approach we adjust the stiffness of a biarticular spring based on the leg force. Therefore, there is no need to track desired impedance with the controller. Instead, a sensory feedback circuit can be used to measure the leg force and modulate the hip actuator stiffness. The control loop will be closed at a higher level when this modulation finally influences the leg force. Hence, we do not use a desired signal for tracking. This is similar to reflex-based control in human locomotion that results in stable gaits without direct control of the target states (Geyer et al., 2003; Geyer and Herr, 2010).

In Zhao et al. (2017), we have implemented the FMCH control approach on LOPES II exoskeleton through separate control of the knee and hip torques using Equation (12). Since there was no physical spring in that rigid exoskeleton, the joint angles are measured to emulate virtual springs. With this approach, the activation of different muscles was decreased and more than 10% reduction in metabolic cost was achieved compared to transparent mode control. As there was no biarticular muscle in the LOPES II robot, we could not benefit from the other properties of biarticular actuation such as transferring energy and synchronization between the adjacent joints. Therefore, using biarticular thigh actuators in a soft suit may be beneficial to achieve higher performance with the assistive device using variable impedance actuators. One possible type of actuator for implementing the proposed method is a pneumatic air muscle (PAM).

In LOPES experiments, the results are compared with transparent mode in which the control target is zeroing the interaction force between the robot and human body. Therefore, there is no additional mass in the assisted mode with FMCH-based control compared to the transparent mode. In addition, the robot can generate force in both directions and not just pull as in exosuits. Hence, 13% reduction in metabolic cost using only one of the thigh biarticular muscles in our simulations is an achievement compared to 10% reduction with LOPES II via bidirectional actuators. This demonstrates the potential advantages of employing soft-suits and biarticular compliant actuators (e.g., PAMs). With biarticular actuators it is possible to avoid internal losses by transferring energy between two joints instead of positive work at one joint and negative at the adjacent joint. Furthermore, an adaptation of human motor control to the robot can even increase the efficiency of the proposed approach.

In addition to more aligned force direction and lower inertia of the soft suits, their lighter weights are of advantage compared to rigid exoskeletons (Panizzolo et al., 2016). In this paper, we showed that an additional weight of the exosuit (about 5% of the body weight) close to body CoM does not have substantial effects on assistance. It is of utmost significance that these wearable robots can appropriately interact with the body (Ding et al., 2017). In contrast to the exoskeletons with actuators paired with the biological joints, softsuits are merely capable of generating tensile forces, that prevents resistance against natural walking, and hence provides comfort and reduced metabolism (Asbeck et al., 2014). Based on our control method, the mechanism can be simply adjusted to individuals of different body constitutions and motor control properties. In Ding et al. (2018), Bayesian optimization was used to identify the peak and offset timing of hip extension assistance that minimizes the energy expenditure of walking with a wearable device. Similar learning based methods can be easily applied to our method to find the variable spring parameters (*k*_*h*_ and ψ_0_) for each subject. It means that instead of time-based optimization, reflex-based control is employed and minimal parameter space is sufficient for finding the optimal controller. Using PAMs with adjustable compliance for actuation in the soft suit, the only required sensory information for control is the leg force. Therefore, the proposed mechanism for the soft suit can be implemented using minimal sensory measurements.

**Figure 12:**
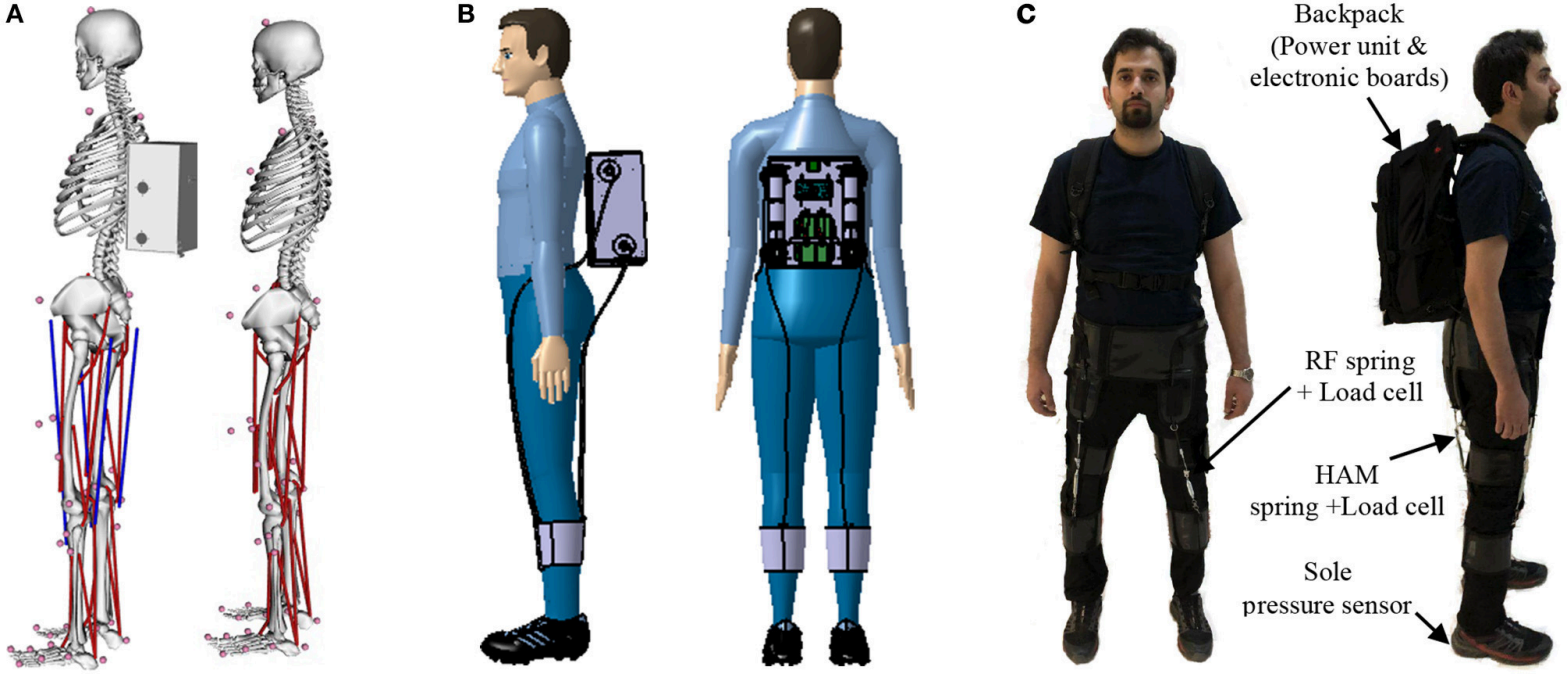
Further implementation of the proposed approach on exosuit. **(A)** OpenSim model with and without assistive device. Red lines show unassisted muscles and blue lines show the assisted ones. **(B)** Design of the exosuit in Catia including 4 motors to actuate biarticular thigh muscles. **(C)** Picture of the manufactured exosuit, the subject gave permission for the publication of this image.

One drawback of the neuromuscular control model used in this study is the lack of rectus femoris. This muscle was neglected due to its minor contribution in normal walking. In order to investigate the idea of assisting human locomotion based on the proposed approach we have implemented the same mechanism using an OpenSim model of human walking. We achieved similar results in reduction of muscle activation and total metabolic costs with this model. One main issue with OpenSim models was that changing the structure of the model requires an adaptation of the control. This feature is provided in the reflex-based neuromuscular models, but in the OpenSim model, it is missing. This means that better results could be achieved by further tuning the model. Finally, we have built an exosuit (Figure 12), which works based on the biarticular actuation of thigh segment. In future we will implement the proposed controller on this system. The hardware properties of the exosuit (with mass) used in both models (Geyer model and OpenSim) are borrowed from this recently manufactured wearable robot. In this design, we focused on thigh biarticular actuators to validate the FMCH-based control methods on a soft-exo. This design principle makes this assistive device different from the other previously developed exosuits (Asbeck et al., 2014; Ding et al., 2017).

The bioinspired balance control based on the VPP concept is supporting efficient locomotion with reduced CoM accelerations and decelerations during the gait cycle. This control concept of using leg force feedback for control of the muscles could be extended for the ankle joint. However, due to the asymmetric function of the human foot during locomotion, the function of the individual ankle muscles on supporting body against gravity and in maintaining balance is still not well understood. Additional research will be required to better understand the interplay between hip and ankle strategies for stable locomotion while keeping the body aligned upright.

## Author contributions

MS is the main and corresponding author of the article. He was responsible for the conception and design of simulations and exosuit design, analysis and interpretation of results and writing of the manuscript. HB implemented the methods on neuromuscular model, performed simulation studies and provided the results. He has also a contribution in writing and revising the paper and discussions. MI was involved in discussions, writing the paper, design and manufacturing the exosuit. AS was involved in conception, description of the results, discussions, and revising the paper.

### Conflict of interest statement

The authors declare that the research was conducted in the absence of any commercial or financial relationships that could be construed as a potential conflict of interest.
