# Supplementary material for: Leg Force Control Through Biarticular Muscles for Human Walking Assistance
Source: Front Neurorobot. 2018 Jul 11;12:39. doi: 10.3389/fnbot.2018.00039 (PMC6050398; doi:10.3389/fnbot.2018.00039)
Supplement: Supplementary file 1 [file Data_Sheet_1.docx]

# Appendix

Relation between hip biarticular muscle length and the angle between the trunk and the virtual leg that was shown in Eq (10) is proved here. According to Fig. 11, assume the knee and hip angles are changing with $\Delta\varphi_{k}$ and $\Delta\varphi_{h}$, respectively. Then, the muscle length change $\Delta l$ is calculated as follows.

$\Delta l=r_{h}\Delta\varphi_{h}-r_{k}\Delta\varphi_{k}$ 

In which $r_{k}$ and $r_{h}$ are the lever arms at knee and hip, respectively. Considering lever arm ratio between hip and knee equal to 2, this equation results in

$\Delta l=r_{h}(\Delta\varphi_{h}-\frac{\Delta\varphi_{k}}{2})$ 

As shown in Fig. 11, the virtual hip angle $\psi$ is given by

$\psi=\varphi_{h}-\frac{\varphi_{k}}{2}$ 

This relation obtained because of the same length of the thigh and the shank segments. Therefore, by finding the changes in the virtual hip angle based on knee and hip angle changes and replacing in Eq (17) the muscle length change will be given by the following equation, which is equal to Eq (10).

$\Delta l=r_{h}\Delta\psi$ 


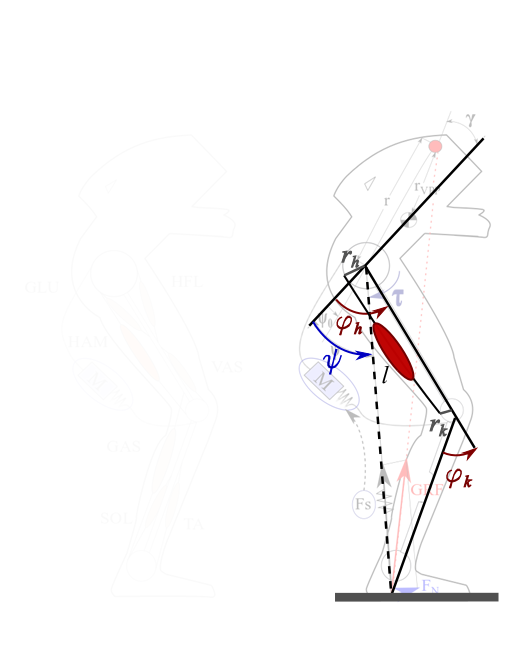


FIGURE 13 The relation between different joint angles, muscle length and virtual hip angle.
